# Amniote phylogeny and the position of turtles

**DOI:** 10.1186/1741-7007-10-64

**Published:** 2012-07-27

**Authors:** S Blair Hedges

**Affiliations:** 1 Department of Biology, Pennsylvania State University, University Park, PA 16802-5301, USA

## Abstract

The position of turtles among amniotes remains in dispute, with morphological and molecular comparisons giving different results. Morphological analyses align turtles with either lizards and their relatives, or at the base of the reptile tree, whereas molecular analyses, including a recent study by Chiari *et al. *in *BMC Biology*, place turtles with birds and crocodilians. Molecular studies have not wavered as the numbers of genes and species have increased, but morphologists have been reluctant to embrace the molecular tree.

Please see Research article www.biomedcentral.com/1741-7007/10/65

## Commentary

**Figure 1:**
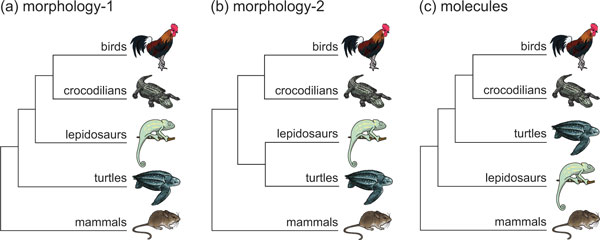
**The three competing theories for the evolutionary position of turtles among amniote vertebrates**. **(a) **Morphology-1 (turtles early): the classic morphological hypothesis, with turtles branching early. **(b) **Morphology-2 (turtles with lepidosaurs): another morphological hypothesis, which groups turtles with lizards and their relatives. **(c) **Molecules (turtles with archosaurs): the molecular hypothesis, which groups turtles with birds and crocodilians.

The tree of life is rapidly coming into focus as hundreds of molecular phylogenies are published each year. For the most part, trees from morphology and molecules have agreed, but there are some notable exceptions, with one being the position of turtles. Classically, the absence of temporal openings in the skull of turtles, the anapsid condition, has been used as evidence to place turtles at the bottom of the amniote tree, after the single-holed (synapsid) mammals split off but before the double-holed (diapsid) reptiles diversified [1] (Figure 1a). Those diapsids include the lepidosaurs (lizards, snakes, amphisbaenians, and tuataras), crocodilians, and birds. Some morphologists have agreed with the classical position of turtles [2] whereas others have interpreted data differently, finding that turtles group with lepidosaurs [3] (Figure 1b). In contrast, virtually all molecular studies, including a recent one in this journal, have found turtles to group with birds and crocodilians, the archosaurs [4-7] (Figure 1c). Although a few morphological characters support a turtle-archosaur group [8], morphologists in general have not embraced the molecular tree.

This dispute is similar to other major controversies in amniote evolution, such as the relationships of squamate reptiles (lizards, snakes, and amphisbaenians) [9] and African mammals (Afrotheria) [10]. However, resolution of those controversies has varied. For example, the molecular tree of squamates has some support from morphological characters, and is slowly gaining hold among evolutionary biologists, but not without resistance. Few morphological characters support Afrotheria, but the inferred biogeographic story, involving continental breakup in the Cretaceous, is so compelling that it quickly gained wide acceptance. This suggests that corroboration from independent evidence, such as biogeography, can go a long way toward resolving a conflict. However, no broad consensus has emerged in the case of turtles. Morphological data and molecular data remain at odds, and biogeographic support for the position of turtles is lacking, probably because turtles originated on the supercontinent Pangaea, before it broke apart. The position of turtles, therefore, represents a classic example of conflict between molecules and morphology.

The study of Chiari *et al. *[7] raises the molecular bar even higher. They analyzed DNA sequence data from 248 genes in 14 amniotes, subjecting those data to a battery of phylogenetic analyses designed to overcome potential biases. The result was, once again, significant support for a close relationship of turtles and archosaurs. A separate study published last year, involving thousands of genes from the transcriptome but fewer species, obtained the same result [6]. These two studies are the largest yet to address the higher-level relationships of amniotes and demonstrate that the molecular position of turtles (Figure 1c) is unwavering.

In systematics there is usually a trade-off between the number of characters (for example, nucleotide sites) and taxa (for example, species), and even these two large molecular studies could be viewed as having too few taxa. The authors of both studies encountered some systematic biases during their analyses, likely attributable to limited taxonomic sampling. This means that there is room for improvement in the future. For example, the earliest-branching lepidosaurs (tuataras) and birds (paleognaths) would be important additions to large molecular data sets, to help stabilize the phylogenies.

One molecular study using micro-RNAs, published recently, stands out among all others in concluding that turtles are most closely related to lepidosaurs, thus supporting morphology [11]. At face value, this could be viewed as reconciling the lengthy dispute in favor of morphology, or at least muddying the waters. But on closer inspection, only a single character supports two key nodes in that tree (reptiles and archosaurs), making the result non-significant and thus not a challenge to the molecular position of turtles. The usefulness of micro-RNAs for tree-building has yet to be established.

On the morphological side, there is mostly agreement that turtles are diapsids [8], albeit closer to lepidosaurs (Figure 1b) than to archosaurs, which is in better agreement with molecules than the classical position. Also, the classical position (Figure 1a) requires a lengthy gap in the fossil record leading to turtles, which is hard to explain for a vertebrate group that is known to fossilize well. In either case, the morphological evidence cannot be dismissed because it is critical for understanding relationships of the major groups of amniotes, such as pareiasaurs, aetosaurs, and ichthyosaurs, among others, that are long extinct. Moving turtles to another location in the tree, with archosaurs, would cause reinterpretations of character evolution, probably impacting the evolutionary position of extinct groups.

Fortunately, major conflicts between molecules and morphology, like this one involving turtles, are not common in evolutionary biology. They are usually resolved relatively quickly as new data are collected and analyzed, or independent evidence such as biogeography is brought to bear on the issue. In this case, determining which result is correct will require additional evidence, such as greater taxon-sampling in molecular data sets and greater scrutiny of the morphological and fossil evidence. In the end, having a stable amniote tree that includes turtles and fossil groups will be of tremendous value in understanding the ecological, physiological, and biogeographic history of amniotes and their adaptive radiation on land and in the seas.
